# Exploring post-COVID-19 mental health disorders: a study protocol for the adult population of Tirana

**DOI:** 10.1192/j.eurpsy.2025.2024

**Published:** 2025-08-26

**Authors:** L. Zikaj, V. Qemalli, G. Qirjako, E. Roshi, S. Qirjako

**Affiliations:** 1 Lab Department, Polyclinic of Specialties No.3; 2 Psychiatry department, Community Mental Health Center No. 3; 3 Department of Public Health; 4 University of Medicine, Tirana, Albania

## Abstract

**Introduction:**

The COVID-19 pandemic has emphasized the crucial need to understand the lasting mental health effects on individuals.

**Objectives:**

This study aims to investigate the occurrence and connections of post-COVID-19 mental health disorders among adults treated at a community mental health center in Tirana. By closing existing knowledge gaps, it seeks to lay the groundwork for tailored interventions.

**Methods:**

Employing a historical cohort study design, data will be collected from Tirana residents diagnosed with COVID-19. Comprehensive clinical and laboratory assessments will focus on mental health outcomes post-infection. Demographic, socio-economic, psychiatric, and biomarker data will be analyzed to provide a thorough understanding of post-COVID-19 mental health disorders in the study cohort.

**Results:**

Preliminary findings are expected to provide valuable insights into the occurrence and connections of post-COVID-19 mental health disorders among Tirana’s adult population. Statistical analyses will identify potential risk factors, informing the development of interventions. Robust data presentation will enhance the credibility and applicability of the study outcomes.

**Conclusions:**

This study promises to elucidate the public health implications of post-COVID-19 mental health disorders in Tirana, guiding targeted interventions. Recommendations based on study findings aim to strengthen mental health services and implement tailored interventions, addressing the unique needs of the community.

Main Messages:

Assessing post-COVID-19 mental health disorders in Tirana informs targeted interventions.

Study findings guide public health actions to enhance mental health services in the community.

*Key words*: Post-COVID-19 mental health disorders; Tirana; community mental health center

**Disclosure of Interest:**

None Declared

